# Exploring the Use of Waste Marble Powder in Concrete and Predicting Its Strength with Different Advanced Algorithms

**DOI:** 10.3390/ma15124108

**Published:** 2022-06-09

**Authors:** Kaffayatullah Khan, Waqas Ahmad, Muhammad Nasir Amin, Ayaz Ahmad, Sohaib Nazar, Anas Abdulalim Alabdullah, Abdullah Mohammad Abu Arab

**Affiliations:** 1 Department of Civil and Environmental Engineering, College of Engineering, King Faisal University, Al-Ahsa 31982, Saudi Arabia; mgadir@kfu.edu.sa (M.N.A.); 218038024@student.kfu.edu.sa (A.A.A.); 219041496@student.kfu.edu.sa (A.M.A.A.); 2 Department of Civil Engineering, COMSATS University Islamabad, Abbottabad 22060, Pakistan; waqasahmad@cuiatd.edu.pk (W.A.); sohaibnazar@cuiatd.edu.pk (S.N.); 3 MaREI Centre, Ryan Institute, School of Engineering, College of Science and Engineering, National University of Ireland Galway, H91 HX31 Galway, Ireland; a.ahmad8@nuigalway.ie

**Keywords:** waste, concrete, marble powder, compressive strength, machine learning algorithms

## Abstract

Recently, the high demand for marble stones has progressed in the construction industry, ultimately resulting in waste marble production. Thus, environmental degradation is unavoidable because of waste generated from quarry drilling, cutting, and blasting methods. Marble waste is produced in an enormous amount in the form of odd blocks and unwanted rock fragments. Absence of a systematic way to dispose of these marble waste massive mounds results in environmental pollution and landfills. To reduce this risk, an effort has been made for the incorporation of waste marble powder into concrete for sustainable construction. Different proportions of marble powder are considered as a partial substitute in concrete. A total of 40 mixes are prepared. The effectiveness of marble in concrete is assessed by comparing the compressive strength with the plain mix. Supervised machine learning algorithms, bagging (Bg), random forest (RF), AdaBoost (AdB), and decision tree (DT) are used in this study to forecast the compressive strength of waste marble powder concrete. The models’ performance is evaluated using correlation coefficient (R^2^), root mean square error, and mean absolute error and mean square error. The achieved performance is then validated by using the k-fold cross-validation technique. The RF model, having an R^2^ value of 0.97, has more accurate prediction results than Bg, AdB, and DT models. The higher R^2^ values and lesser error (RMSE, MAE, and MSE) values are the indicators for better performance of RF model among all individual and ensemble models. The implementation of machine learning techniques for predicting the mechanical properties of concrete would be a practical addition to the civil engineering domain by saving effort, resources, and time.

## 1. Introduction

Iran, Italy, China, Turkey, India, Egypt, Spain, Brazil, Algeria, Sweden, and France are the main marble-producing countries [1,2,3,4]. India is the third most marble-producing country around the globe, and almost 10% of the worldwide marble powder is quarried here [5]. In addition, the import and processing of stone are majorly done in countries such as Pakistan, the United States, Egypt, Saudi Arabia, Portugal, Germany, France, Norway, and Greece [6]. During different stages of stone mining and processing procedures, a bulk quantity of marble waste is generated. Out of which, up to 60% is generated as a result of marble quarrying only [7]. Marble dust in finer form that is produced as a result of its sawing and cutting can cause harmful health issues. Furthermore, the dumping of this marble dust can result in poor soil properties and the fertility reduction of respective land [8]. Almost 30% of marble waste is produced during the working of marble stone [9]. The global annual production of marble and granite was nearly 140 million tonnes in 2014, as per USGS [10]. There were approximately 2 billion tonnes of marble resources in India only, as of April 2015, as per the UNFC system. Only 0.23% were reserved resources, and 99.77% were under the remaining resources category [11]. In 2015, China produced around 350 million sq. meters of marble planks, depicting China as World’s largest marble producer [12]. Chauhdary [13] reported the availability of almost 160 million tonnes of marble reserves and around 2 billion M.T granite reserves in Pakistan as of 2006. In the mining industry of Iran, there were approximately 4.8 million tonnes of raw and/or semi-processed stone in the year 2012–2013 from a total of 473 quarries of marble stone [14]. Egypt used to export nearly 13 lac tonnes of stones annually as unprocessed and processed stones. From Shaq Al–Thoban industrial/site areas of Egypt, nearly 7 lac tonnes waste is generated annually [15]. As far as the marble reserves of Turkey are concerned, these are around 3.8 billion cubic meters [16]. In Turkey, Binici, et al. [17] reported an emerging threat to agriculture and health in the form of marble wastes usually left in situ or settled by sedimentation. Approximately 47 thousand tonnes of solid waste powder is collected annually from quarries in Jordan every year [18]. The same is the case with Spain and some other countries [19]. In past years, the marble powder is usually used in mortar, concrete, tiles, cement, embankments, and pavements [20], in addition to the desulfurization process, soil stabilization, ceramics, and asphalt and polymer-based composites [21]. In Italy, a group of researchers also developed a consortium to rehabilitate and restore the Oresei marble chain in Sardinia. This chain was being exploited for quarrying and landfilling [22]. As per the definition of sustainable development by Brundtland [23], keeping in mind the environmental perspective, the addition of mineral admixtures and different waste materials has gained much importance with the aim to reduce the consumption of natural resources. However, the natural resources consumption for the production of concrete is still inevitable. In addition, the extraction of local natural resources within limited surrounding region is unable to meet the said needs; thus becoming un-sustainable in near future. Accordingly, the usage of waste materials in concrete production should be promoted in construction sector. In addition, the alternative sustainable approaches should also be introduced for reducing the consumption of natural materials at national as well as international level [24,25,26,27]. Whereas, at local level, recycled aggregates are usually used for road materials stabilization. This is a rare approach due to the less feasible crushing process with respect to traditional approach. The extraction of natural resources is required in traditional approach. Bottom ash and marble dust (MD) are some locally and abundantly available by-products that are usually treated as waste materials and thus ultimately causing environmental pollution.

On a rough estimate, the global annual concrete production is approximately 25 billion tons. Concrete has a very low embodied energy and carbon footprint compared to other building materials. However, due to its wide use in many applications, concrete production has a considerable carbon footprint, contributing to 8% of global carbon dioxide emissions [28,29]. Globally, concrete production accounts for 7.8% of nitrogen oxide emissions, 4.8% of sulfur oxide emissions, 5.2% of particulate matter emissions smaller than 10 mm, and 6.4% of particulate matter emissions smaller than 2.5 microns [30]. It is worth noting that only half of the cement is used in concrete [31], and the remaining is used in blocks, mortar, and plaster [32]. Nonetheless, due to the widespread use of concrete in modern civilization, concrete production accounts for a significant portion of global CO_2_ emissions through construction [32]. Aiming toward sustainable development, the usage of environment-friendly by-products is considered an effective strategy toward reducing CO_2_ emissions [31,33,34,35]. Marble dust (MD), having abundant availability in Turkey, China, Iran, Italy, and India, is also an alternative which can be used as a replacement for cement in the production of concrete. Marble, due to its durable properties, is usually used in multiple non-structural applications such as cladding, floors, architectural decoration for indoors and sculpture etc. Considerable waste is generated during the shaping and cutting processes of various marble applications in the form of dust particles. These materials are contaminating the natural resources in terms of environmental damage. Partial replacement of cement and other constituents of concrete has already been made extensively by industrial by-products in various studies [36,37,38,39,40,41,42,43,44]. The reuse of MD, due to its chemical nature, in the production of concrete came out to be an alternative sustainable approach. The use of MD, either as a natural aggregate [9,45,46] or as a replacement for Portland cement (PC) [16,47,48,49], has been studied in various research. Generally, MD has been used as up to 60% replacement in different forms. Gesoğlu, et al. [50] reported a 20% decreased slump due to MD as a PC replacement. Concrete having MD showed similar consistency with respect to reference mix as reported by Seghir, Mellas, Sadowski, and Żak [4]. Contrary to this, Alyamac, Ghafari, and Ince [19] stated that the incorporation of MD in concrete improved its fresh properties. In addition, the strength of concrete having MD is still questionable. Topcu, et al. [51] reported the decreased compressive strength with an increase in MD content. The same behavior was also reported by Gencel, et al. [52]. The 5% of MD replacement in concrete production came out to be an optimum content for compressive strength, as reported in several studies [50,53,54]. However, Li, et al. [55] reported the same with 10% MD replacement in concrete. Li, Huang, Tan, Kwan and Liu [12] and Li, Huang, Tan, Kwan and Chen [55] also proposed a paste replacement method for reducing significant (i.e., 33%) cement content and enhancing the utilization of MD waste, having enhanced durability and strength. Seghir, Mellas, Sadowski and Żak [4] reported an enhancement of marble powder porosity by 15% in result of reduced hydration products. The major focus of existing studies is on replacement of cement with alternative sustainable materials for reduction in emissions, caused by PC. Marble waste is used as cement replacement in concrete by various researchers [9,46,50,52,54,56]. Rodrigues, De Brito and Sardinha [46] investigated the incorporation of marble dust having 5, 10, and 20% content as cement replacement in concrete. The study reported positive effect on compressive strength of concrete with cement replaced up to 10% marble dust; however, reduced compressive strength is observed in concrete having 25% of marble dust. The compressive strength is reduced by 13.46% with 20% marble dust content, as reported by Gesoğlu, Güneyisi, Kocabağ, Bayram and Mermerdaş [50]. Another study reported decrement in compressive strengths by 91%, 86%, and 76% having cement replaced by 20%, 30%, and 40% marble dust contents, respectively [52]. Şanal [57] reported enhancement of pore structure due to an increase in the capillary structure of concrete by adding 10% marble dust as cement replacement, ultimately resulting in reduced mechanical properties of concrete.

Concrete is the second most widely used commodity around the globe [58]. Due to its multiple properties such as strength, stiffness, density, fire/thermal resistance, porosity, and durability, concrete is being most commonly used as a building material all around the world. Compressive strength is the most dominating factor among all these, as it directly affects the durability of concrete [59,60]. Concrete is a heterogeneous material constituted by cement, sand, aggregates, and water, as it has different compressive strength values [61]. All the ingredients mentioned above and respective mixtures affect the compressive strength of concrete in terms of water/binder ratio, aggregate size, binder type, or waste composition [62]. The compressive strength of concrete is hard to predict precisely due to its complicated mixture. The determination of concrete compressive strength can be made in the laboratory by crushing standardized cylinders/cubes after specified curing post to the casting of samples [63]. This is globally a standardized method. However, as a result of advancements in technological development, laboratory tests are now insufficient and uneconomical due to the involved time and cost. Nowadays, due to the artificial intelligence (AI) evolution, mechanical properties of concrete can also be predicted by using machine learning (ML) algorithms [64,65,66]. ML techniques such as classification, clustering, and regression, can be used to estimate various parameters along with varied efficiency and can also help in predetermining the accurately précised compressive strength of concrete.

The performance prediction of various parameters using machine learning algorithms is known for many years. As far as the field of civil engineering is concerned, this trend is increased significantly in the past few years. It is because of the highly accurate prediction of mechanical properties (Table 1). The working principle of machine learning is the same as that of conventional algorithms high accuracy of nonlinear behavior with respect to the linear one. Artificial neural networks (ANN), support vector machines (SVM), decision trees (DT), gene expression programming (GEP), random forest (RF), and deep learning (DL) are widely used prediction techniques in case of mechanical properties of concrete [67]. The shear strength of steel fibers reinforced concrete beams was predicted with the help of eleven algorithms by Rahman, et al. [68]. ANN with optimizer as multi-objective grey wolves (MOGW) was used by Behnood and Golafshani [69] for predicting the static properties of silica fume modified concrete. Güçlüer, et al. [70] used ANN, DT, LR, and SVR to predict the compressive strength of concrete. The tensile strength and compressive strength of waste concrete were predicted with ANN algorithm by Getahun, et al. [71]. Ling, et al. [72] used SVM to predict concrete compressive strength in marine and the results were compared with that of DT and ANN models. Yaseen, et al. [73] also used different ML approaches for the prediction of load carrying capacity, under compression, of light-weight foamed concrete. A machine learning algorithm was also used by Taffese and Sistonen [74] for assessing reinforced concrete structures’ durability. Yokoyama and Matsumoto [75] developed an automatic crack detector for concrete structures using machine learning. Concrete samples photographs were used for learning data, whereas deep learning was applied for crack detection. The accuracy level of ML models was determined by Chaabene, et al. [76]. Ahmad, et al. [77] performed ensembled machine learning (EML) and standalone techniques for the prediction of concrete’s compressive strength and accuracy comparison. It is reported that the outcome predicted from EML techniques has more accuracy than that by standalone technique. However, the range of standalone technique results was also acceptable. Song, et al. [78] determined the compressive strength of ceramic waste modified concrete both experimentally and with standalone techniques. Marginal variation in experimental results and prediction model’s outcomes was reported. Neural networks and decision trees, which are also called classification trees, are two popular ways to model data. These two models have different ways of modeling data and finding relationships between variables. The nodes in the neural network make it look like the human brain and very complex structure is formed. While the decision tree is an easy way to look at data from the top down. Decision trees have a natural flow that is easy to understand and are also easy for computer systems to program. The data point in decision tree models at the top of the tree has the most effect on the response variable in the model. On the other hand, the visual representation of neural network models does not make it easy to understand the working. For neural network model, it is hard to make computer systems, and it is almost impossible to make an explanation because of complex structure. Therefore, decision tree-based algorithms (AdaBoost and bagging) are considered in the study because these trees are so easy to understand, they are very useful for modeling and showing the data visually without any complex structure. Accordingly, the current study aims the usage of advanced techniques for forecasting the concrete properties.

## 2. Research Significance

The incorporation of waste materials in concrete to improve its mechanical characteristics has been done in various studies. However, the stepwise laboratory procedure, i.e., casting of specimens, curing for a specified time, and testing is still a concern in terms of cost and time. Novel machine learning techniques are being introduced for forecasting the behavior of waste concrete in terms of mechanical properties to overcome the issues mentioned above, i.e., the excessive consumption of time and cost. However, the results of different machine learning models are still inconsistent depending on the type of material, data set, and other contributing input/output parameters. Therefore, this paper aims to investigate marble dust concrete with the intention of marble dust waste management and identify the optimal machine learning technique. The novelty and significance of the current study are to conduct experimentation on waste marble (powder-based) concrete (WMC) and development of WMC prediction model by computational methods. Additionally, this study is focused on predicting and comparing the compressive strength of WMC through supervised ML approaches. The AdB, RF, Bg, and DT approaches are employed to predict and compare outcomes against actual results. Twenty sub-models are developed in EML modelling to have more accuracy in R^2^ value for the optimization. Prediction performance of each technique is done by using these applications. This research is significant for understanding the input parameter’s role and accuracy for the outcomes obtained through ML algorithms. Individual ML and ensemble approaches are also compared against the results obtained from experimental work. The k-fold cross-validation and statistical checks are also used to evaluate the performance of each model. A discussion on the use of marble for sustainable construction is made.

## 3. Experimentation and Data Description

**Figure 1:**
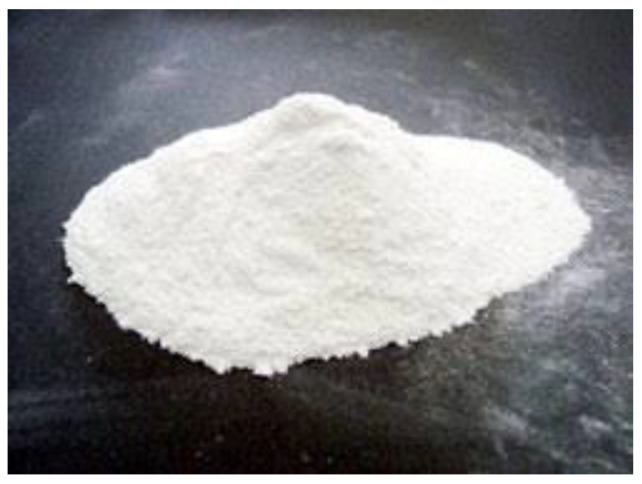
Waste marble powder.

Cement, marble powder, and fine and coarse aggregates are used to prepare 40 mixes. Type-I Ordinary Portland Cement (OPC) is used. ASTM C150 is used to conduct the entire investigation in this research. The chemical composition of used marble and cement is listed in Table 2. The properties of fine aggregate are also determined as per the ASTM standard. Locally available coarse aggregates having a maximum nominal size of 25.4 mm are being used. Furthermore, the physical properties of fine and coarse aggregate can also be seen in Table 3. Marble powder, collected from a local company, is used in this study, as shown in Figure 1. The Blaine fineness value was 2196 m^2^/kg, and the relative density was 2.43 g/cm^3^. The marble powder has a large specific surface area, suggesting that adding it to concretes would improve their cohesiveness.

**Figure 2:**
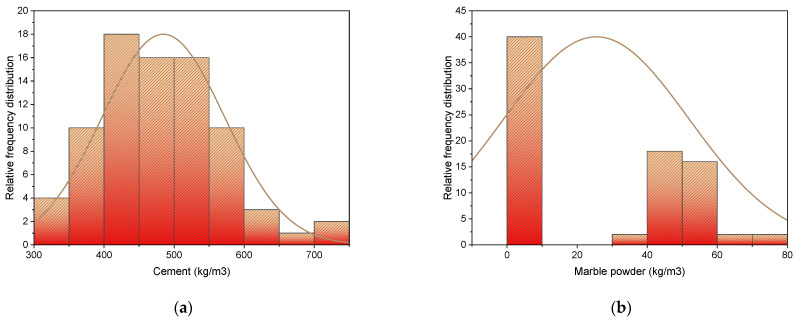

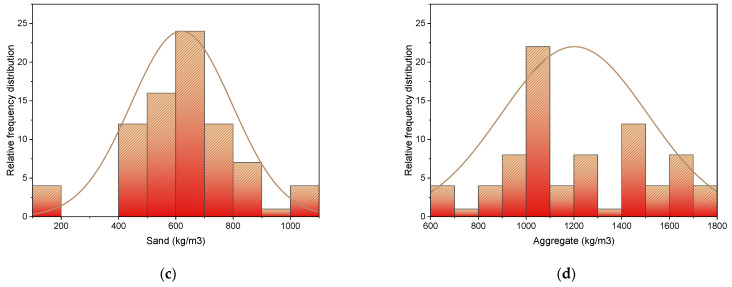
Input parameters relative frequency distribution: (**a**) cement; (**b**) marble powder; (**c**) sand; (**d**) aggregate.

In this study, two different mix designs are considered. Twenty mixes for controlled concrete and twenty for marble replaced concrete are prepared at every 7 days and 28 days. A total of 40 combinations with 240 specimens are prepared (120 in number for each respective day) with a size of 150 mm^3^. De-molding of specimens is done after 24 h, followed by 28 days of water curing. The compression test is performed afterwards, as per ASTM C39, to determine compressive strength. The dataset includes six inputs, i.e., i. cement, ii. marble powder, iii. w/c ratio, iv. coarse aggregates, v. sand and vi. Days for single output, i.e., compressive strength of concrete (refer Table S1 in supplementary materials). The description of statistical analysis regarding input parameters is given in Table 4 and Table 5. Table 4 shows the mean value, the average of the numbers by adding up, and then dividing by total number of values in a dataset. All the parameters are considered in weight units, i.e., kg/m^3^, except for age, which is being considered in days. Brief descriptive coefficients are collected to summarize descriptive statistics to produce a result. Descriptive analysis results are based on input variables data reflecting various information. The minimum and maximum values and ranges for each variable that is used to run the model are also given in tables. However, other analysis parameters, such as standard deviation, mean, mode, and summation of all data points against each variable, are also used for depicting relevant values. Frequency dispersion for every factor that is being utilized in mixes is shown in Figure 2. It has a close connection with distribution probability, a widely used statistics. A relative frequency distribution shows the total observations associated with a class of values or every single value.

## 4. Modelling Techniques Description

**Figure 3:**
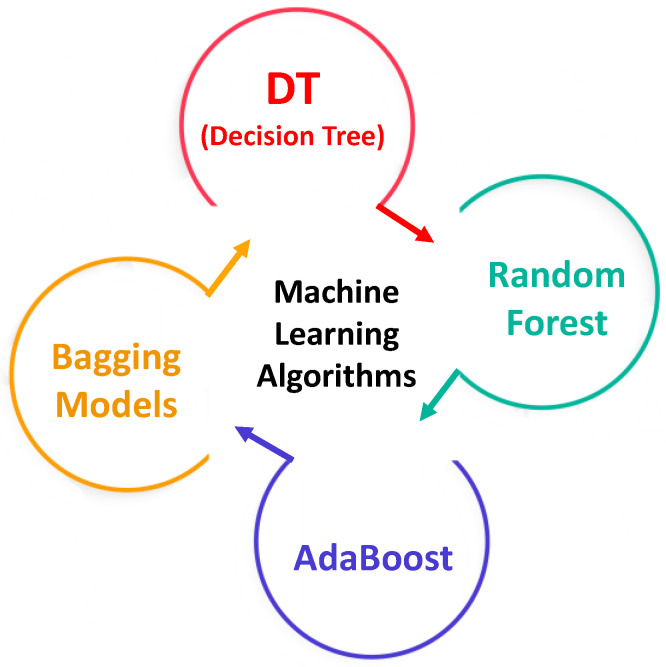
Algorithm flowchart.

Concrete compressive strength prediction algorithms are described in this section. Individual ML (DT) and ensembled ML techniques (i.e., bagging models, random forest and AdaBoost) are employed over Anaconda software by using Python code. Spyder (version 4.3.5) of Anaconda navigator is opted for running the random forest, bagging models and AdaBoost. Such algorithms are usually used to predict required outcomes as per input variables. Six input parameters against one output parameter (i.e., compressive strength) are used for all techniques during the modelling phase. R^2^ values demonstrate the accuracy/validity of all the models. The R^2^ statistic (also named determination coefficients) evaluates the variance response variable as demonstrated by the model fitted against the mean response. It can also be stated as the measurement of how well a model fits this data. 0 value implies the comparison of fitting the mean and model, whereas 1 depicts a perfect fit among data and model. C.S prediction is made with individual, i.e., decision tree, and ensemble algorithm, i.e., bagging models, random forest, and AdaBoost. Figure 3 shows a detailed flowchart of the used algorithm. It may be noted that 50% of data is used for training, and rest of the 50% is used for testing and validation. The error between the experimental and predicted values is also reported for each algorithm, and a discussion is made in Section 6.

### 4.1. Decision Tree Algorithm

**Figure 4:**
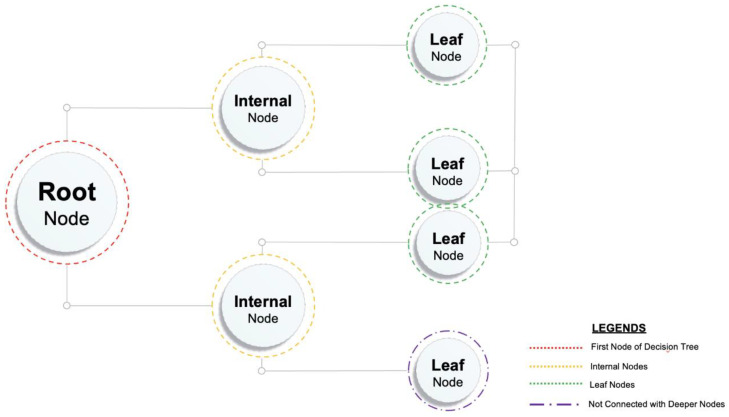
Decision tree schematic diagram.

DT is widely utilized to categorize regression problems and classify difficulties [87]. There are classes within a tree. However, the regression technique is used to predict outcome-independent variables in case of the non-existence of any class [88]. In DT, database attributes are represented by inner nodes. Conclusion rules are denoted by branches, whereas the leaf nodes represent the result. Two nodes, i.e., the decision node and leaf node, are the composition of a DT. Several branches of decision nodes can make a decision, and leaf nodes depicts. Leaf nodes depict the decision’s output, lacking branches. It is named a decision tree as it resembles a tree-like structure that begins with grows as per the number of branches based on a root node [76]. Data samples are bifurcated in multiple segments by DT. An executed algorithm determines the difference between forecasted values and goal at each division point. Errors are also calculated at each division point, and the lowest value variable is selected as a split point for the fitness function, and the same procedure/method is repeated. Figure 4 depicts the decision tree schematic diagram.

### 4.2. Random Forest Algorithm

**Figure 5:**
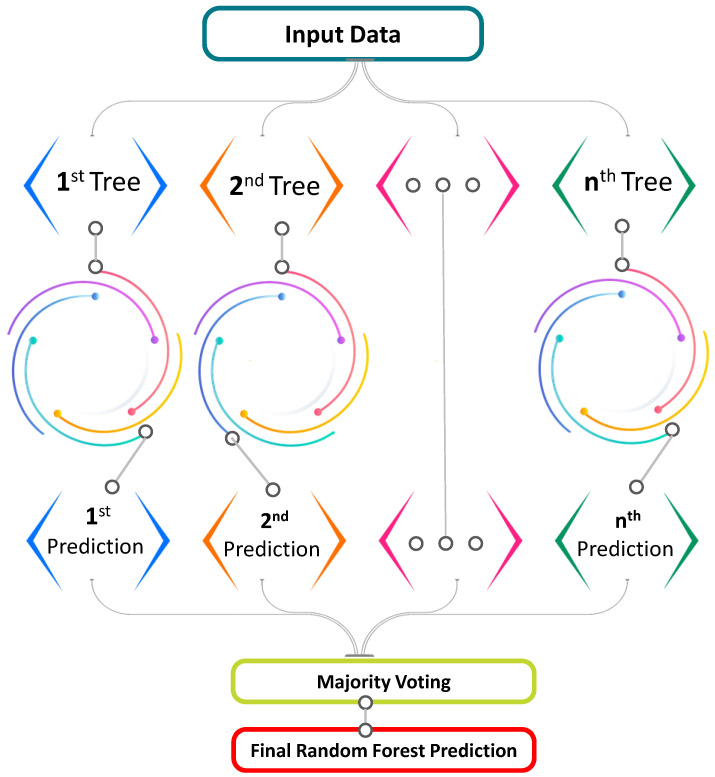
Random forest schematic diagram.

The random forest model is a regression and classification-based approach that has been studied by various researchers till now [86,89]. The compressive strength of concrete is predicted by using the RF model, as done by Shaqadan [90]. The prime difference between RF and DT is the number of trees as shown in Figure 5. A single tree is developed in DT; however, in RF, multiple trees are built that are known as forest. Dissimilar data are selected arbitrarily and accordingly, allocated to respective trees. Each tree has data in rows and columns, and different dimensions of rows and columns are selected. Following steps are carried out for the growth of each tree; the data frame comprises 2/3rd of the whole data that is randomly selected for each tree. This method is known as bagging. Random selection is made for prediction variables, and the node splitting is done by finely splitting these variables. For all trees, the remaining data are utilized to estimate out-of-bag error. Accordingly, the final out-of-bag error rate is assessed by combining errors from each tree. Each tree provides regression, and among all forest trees, the forest with greater votes is selected for the model. The value of votes can either be 1′s or 0′s. Prediction probability is specified by the obtained proportion of 1′s. Among all ensemble algorithms, random forest (RF) is the most sophisticated one. It includes desirable features for variable importance measures (VIMs) with robust overfitting resistance and fewer model parameters. DT is used as a base predictor for RF. Acceptable results can be produced by RF models with default parameter settings [91]. As allowed by RF combinations of parameter settings, and base predictors can be reduced to one.

### 4.3. AdaBoost Algorithm

**Figure 6:**
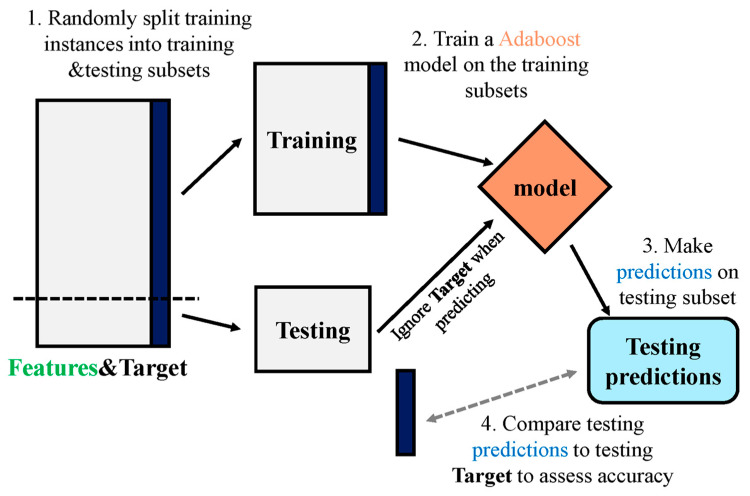
Complete process of prediction via AdaBoost algorithm [93].

Figure 6 shows the entire process of forecasting the AR algorithm outcome. The Ensemble technique is a concept of ML that is utilized for training various models by using a learning algorithm of the same kind [92]. Multiple algorithms are collected, as multi-classifiers, for making an ensemble. A group comprises almost a thousand learners working with the same objective of resolving the issue. Ensemble learning is employed by an AdaBoost algorithm, which is a supervised ML technique. It can also be referred to as adaptive boosting, as weights are re-linked to every instance, with higher weights linked to wrongly classified instances. Boosting techniques are widely utilized to minimize variance and bias in supervised ML. Weak learners can be strengthened by using the said ensemble techniques. Infinite no. of DTs are employed by it for the input data during a training phase. During constructing the initial DT, the erroneously categorized recorded data are prioritized throughout the initial model. Same data records are used only as an input for different other models. The technique mentioned above is repeated till the creation of specified base learners. AdaBoost optimizes enhancement of DTs performance on binary classification issues. In addition, it is also used for enhancing ML algorithms performance. It is specifically effective when it is used with slow learners. These ensemble algorithms are very prevalent in the civil engineering field, especially for predicting concrete mechanical properties.

### 4.4. Bagging Algorithm

**Figure 7:**
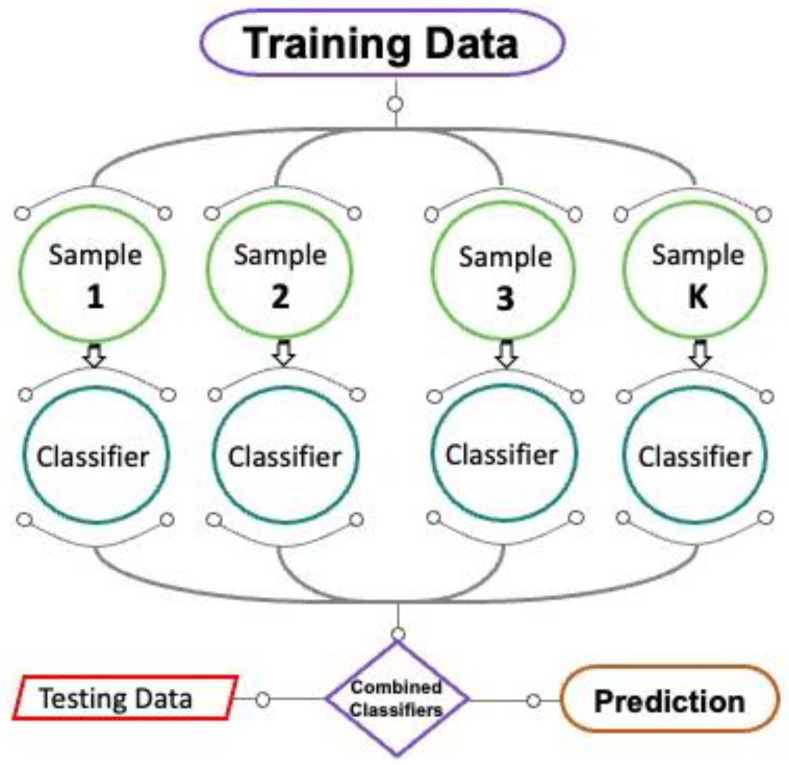
Bagging algorithm flow chart indicating the step-by-step procedure of prediction.

The detailed procedural flow chart of the bagging algorithm is shown in Figure 7. It is basically an equivalent ensemble method that describes the prediction model variance by supplementation with additional data throughout the training stage. The technique of irregular sampling includes the data replacement from a primary set. Employing replaced sampling, every new training dataset can duplicate specific observations. In the procedure of bagging, for every component, there is an equal possibility of appearing in a new dataset. The training set size is not dependent on predictive force. Furthermore, variance may be remarkably declined by precisely tuning the prediction of the desired outcome. Additional models are trained by using these data sets. The mean of predictions by all models is used for this ensemble. In regression, the average of various models’ predictions can be a forecast [94]. A total of twenty sub-models are being utilized for tweaking the bagging algorithm with DT to find the optimized value which produces firm output.

**Figure 8:**
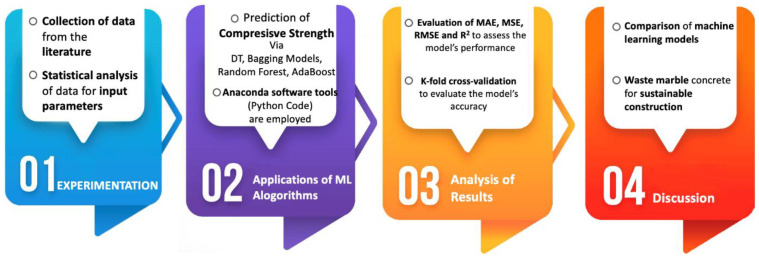
Research methodology.

The flowchart depicting the research approach is shown in Figure 8. Given the three algorithms mentioned above anomaly, further to DT, a combination of ensembles (i.e., AdaBoost, bagging models, and random forest) algorithms is employed in this study for maximizing the respective benefits. Twenty sub-models are employed by ensembled strategies for the determination of ideal value, which develops a firm output. In addition, error evaluations such as mean square error (MSE), mean absolute error (MAE), k-fold cross-validation and root mean square error (RMSE), and statistical checks are made for evaluating the model’s performance. Finally, the comparison of different machine learning models is made, as well as the suitability of waste marble powder in concrete for sustainable construction.

## 5. Experimental Compressive Strength Test Results

**Figure 9:**
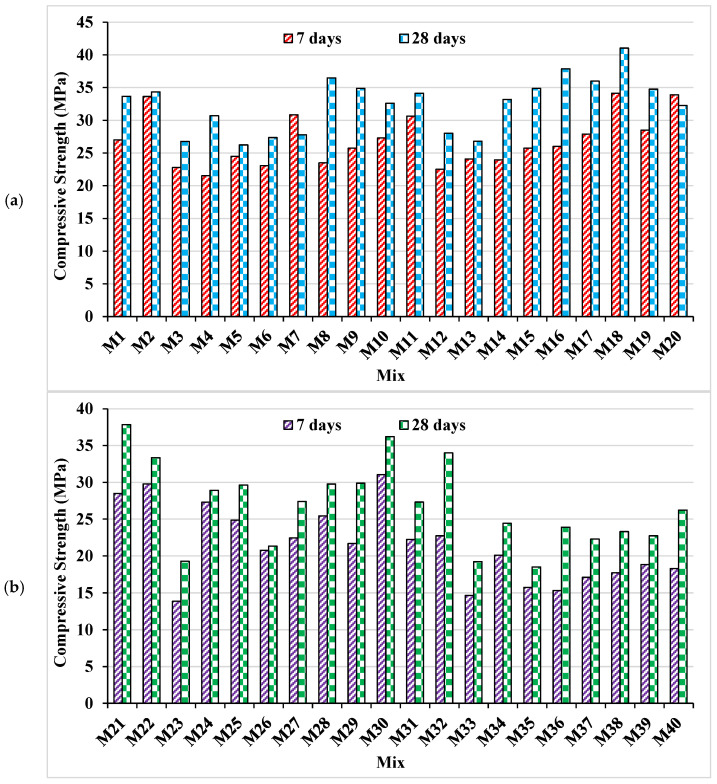
Experimental compressive strength; (**a**) plain concrete; (**b**) marble powder concrete.

From the compressive strength test results, it is identified that a decrement in compressive strength is observed with an increase in the content of marble powder in bricks (Figure 9). The highest C.S at 7-days and 28-days of 34.13 MPa and 41.03 MPa is obtained by M18, which contained 0% marble powder content. Specimens of waste marble powder group achieved a maximum compressive strength of 31.06 and 37.83 MPa at 7 and 28-days, respectively. The maximum decrease in waste marble concrete range is 9.97–48.14%, as compared to 7 days of plain mix. The maximum decrease in waste marble concrete range is 2.9–46.9%, compared to 28 days of plain mix. The increased porosity level with the increase in marble powder content in concrete, and hence the compressive strength is decreased. Şanal [57] reported enhancement of pore structure due to an increase in the capillary structure of concrete by adding 10% marble dust as cement replacement, ultimately resulting in reduced mechanical properties of concrete. This can be caused by the dissimilar C_3_A—tricalcium aluminate content in cement due to its replacement by marble dust [50]. However, in the current study, the worst mechanical property was observed that might result from the increase in the capillary structure of the pores with the addition of marble dust, as reported in the previous study [57].

## 6. Analysis and Modelling Results

### 6.1. Prediction of Compressive Strength by Different Models

- Decision tree modelling

**Figure 10:**
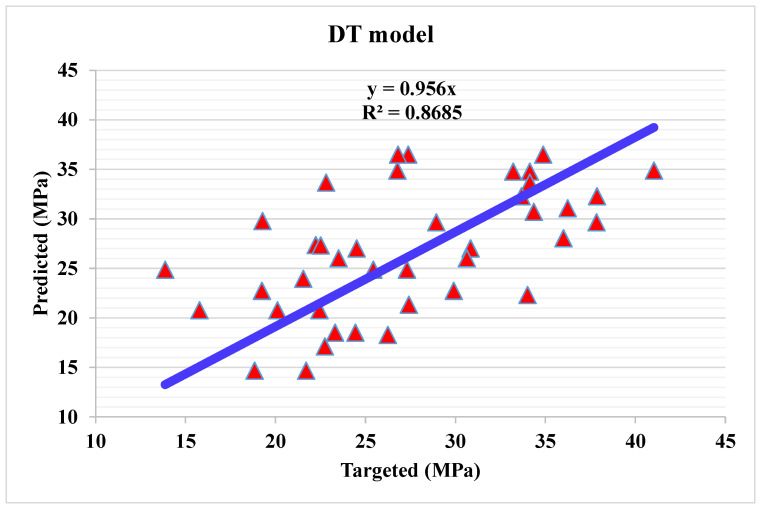
Predicted and actual results of DT model.

**Figure 11:**
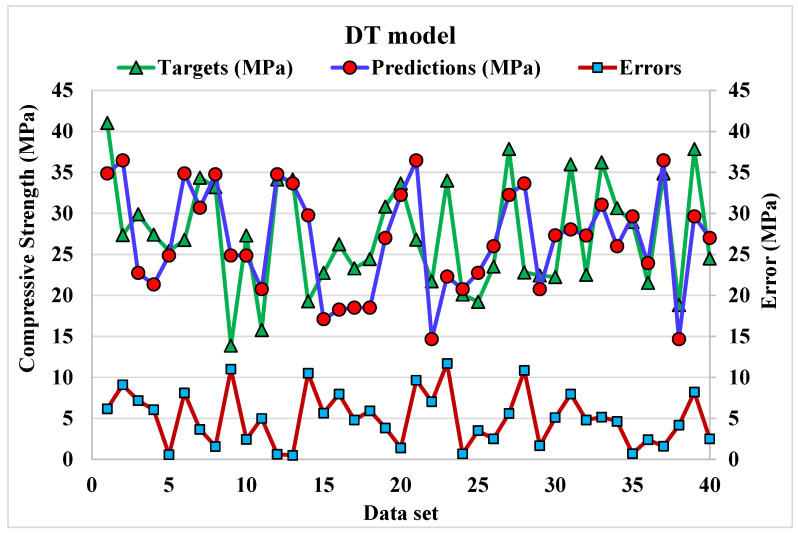
Dispersion of predicted and actual values along with errors for DT model.

Figure 10 depicts a statistical analysis of projected and actual results regarding C.S of WMC for DT modelling. A reasonably précised output and a very low variation between anticipated and actual values can be obtained by DT technique. The accuracy of predicting results can be assessed by having a 0.86 R^2^ value. The blue line represents the correlation between the experimental and predicted values, as evident by the R^2^ value. The higher R^2^ denotes the higher accuracy of the model. The dispersion for predicted and experimental values (targets) and DT model errors is shown in Figure 11. The average, highest, and lowest values of the training set are 6.20, 20.7, and 0.07 MPa, respectively. Whereas 12.5% error values are less than 1 MPa, 37.5% are from 2 to 5 MPa, 32.5% are from 6 to 10 MPa, and 17.5% are higher than 5 MPa.

ii. Random forest modelling

**Figure 12:**
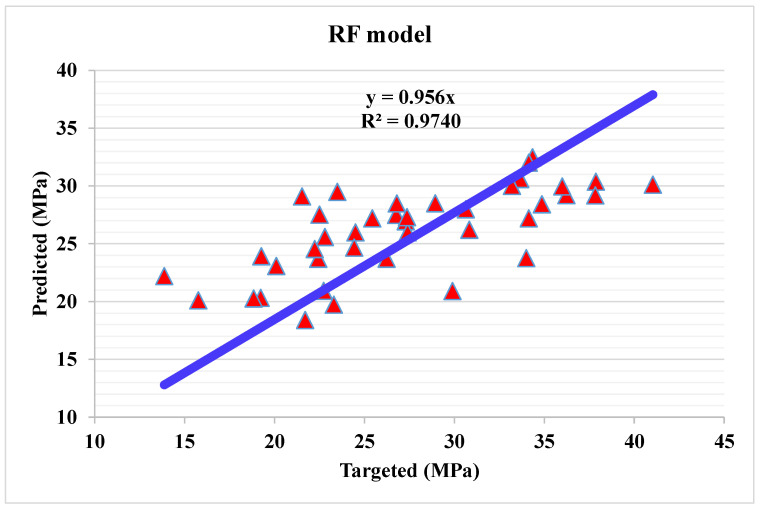
Predicted and actual results for RF model.

**Figure 13:**
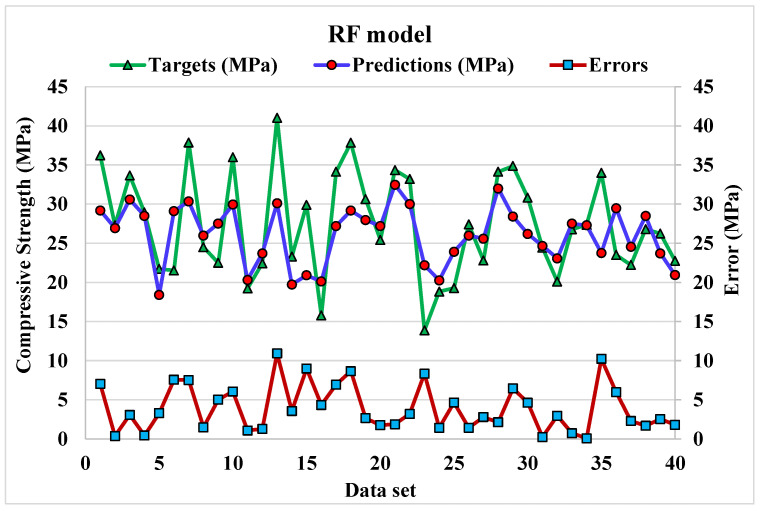
Dispersion of predicted and actual values along with errors for RF model.

The correlation between projected and actual results of RF model is shown in Figure 12. The R^2^ value for the RF model comes out to be 0.97, which represents the highly precise and more accurate of RF w.r.t Bg, DT, and AdB models. Furthermore, the dispersion of projected values, actual targeted values and errors for RF model is shown in Figure 13. The minimum, maximum, and average error values are 0.07, 10.9 and 3.93 MPa. It is noted that 15% of error data are below 1 MPa, 57.5% from 2 to 5 MPa, 22.5% from 6 to10 MPa, and only 5% higher than 10 MPa. This analysis reveals the higher accuracy of RF model w.r.t AdB, DT, and Bg models. It can also be depicted from lower error and greater R^2^ values. In addition, twenty sub-models are employed by EML (Bg, DT, and AdB) to get the optimized value that produces a firm output.

iii. AdaBoost modelling

**Figure 14:**
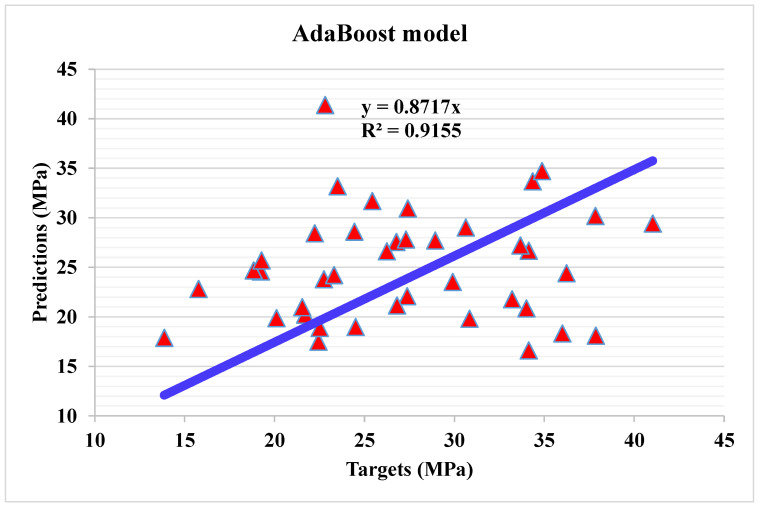
Predicted and actual results for AdaBoost model.

**Figure 15:**
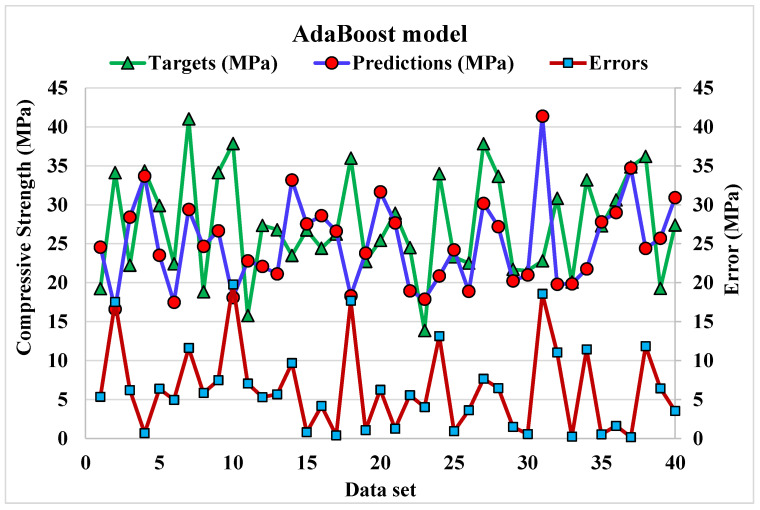
Dispersion of predicted and actual values along with errors for AdaBoost model.

A comparison of projected and actual outputs of AdB model is shown in Figure 14 and Figure 15. The correlation between them is illustrated in Figure 14. The R^2^ value is 0.91, which shows better outcomes when compared to the DT model. The dispersion of actual and predicted values along with errors for AdB model is illustrated in Figure 15. 19.7, 0.15, and 6.34 MPa are the maximum, minimum, and average values for the training set. Whereas 27.5% of error values are below 1 MPa, 20% range from 2 to 5 MPa, 30% range from 6 to 10 MPa, and only 22.5% are higher than 10 MPa. The higher accuracy of AdB model in comparison with the DT model is also depicted by lower error values.

iv. Bagging modelling

**Figure 16:**
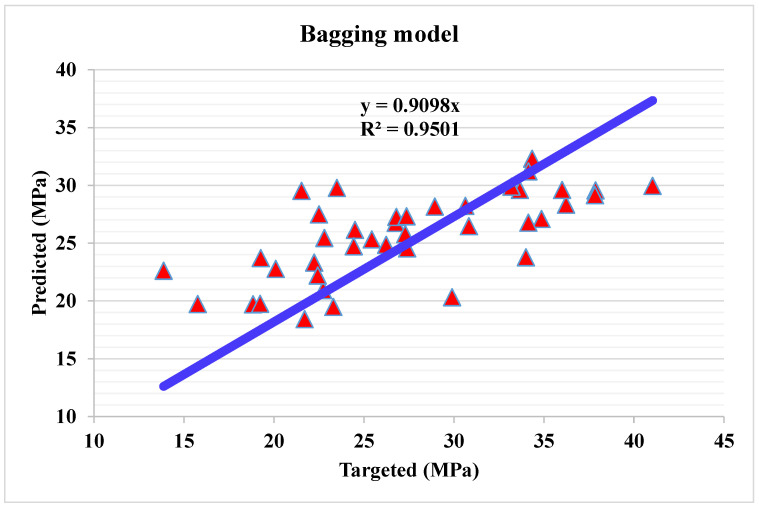
Predicted and actual results for bagging model.

**Figure 17:**
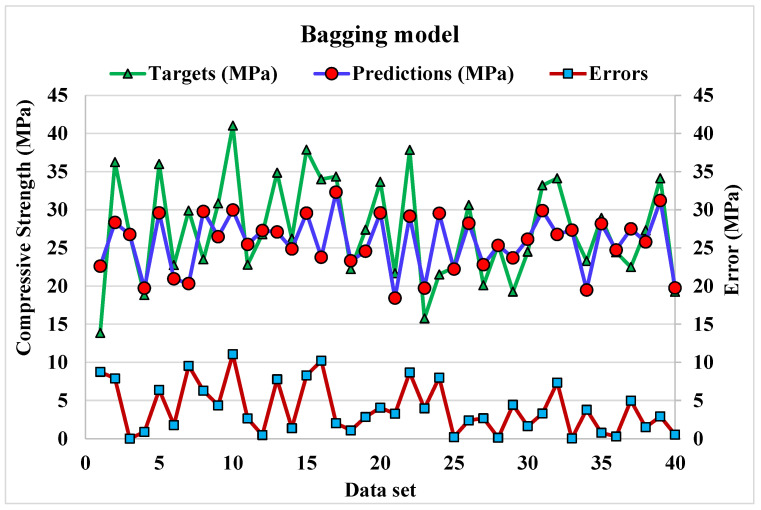
Dispersion of predicted and actual values along with errors for bagging model.

The correlation between predicted and actual output values for Bg model is provided in Figure 16. The R^2^ value for this model comes out to be 0.95, showing considerable accuracy as compared to that of DT and AdB models. The dispersion of actual and predicted values and errors for the Bg model is shown in Figure 17. The maximum, average, and minimum in the training set are 11.07, 3.96, and 0.01 MPa, respectively. Whereas only 25% of error values are below 1 MPa, 45% of values range from 2 to 5 MPa, and 27.5% values range from 6 to 10 MPa. The error distribution and R^2^ are more accurate than that of DT and AdB models for the C.S prediction of WMC. Whereas the R^2^ and error values obtained from all considered ensembled ML models are in an acceptable range, thus depicting better prediction outcomes. Hence, it is observed in this study that EML techniques (RF, AdB and Bg) can predict high accuracy outcomes when compared to standalone DT techniques.

### 6.2. K-Fold Cross Validation Checks

**Figure 18:**
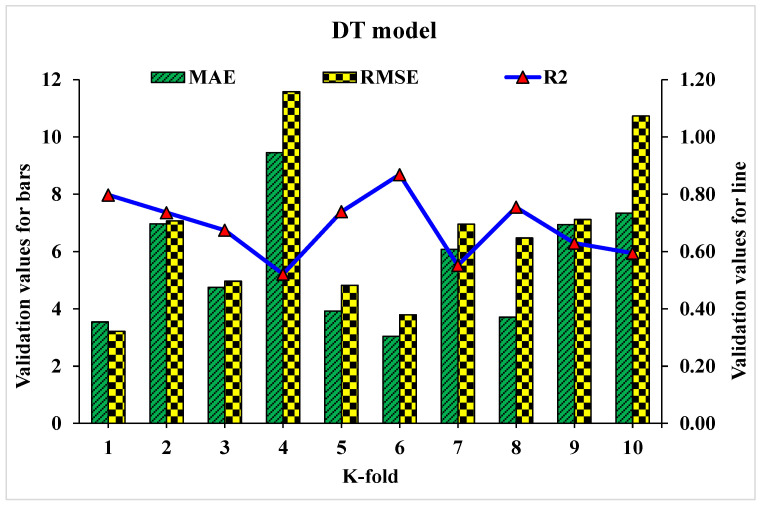
Statistical analysis of DT model for K-fold cross-validation.

**Figure 19:**
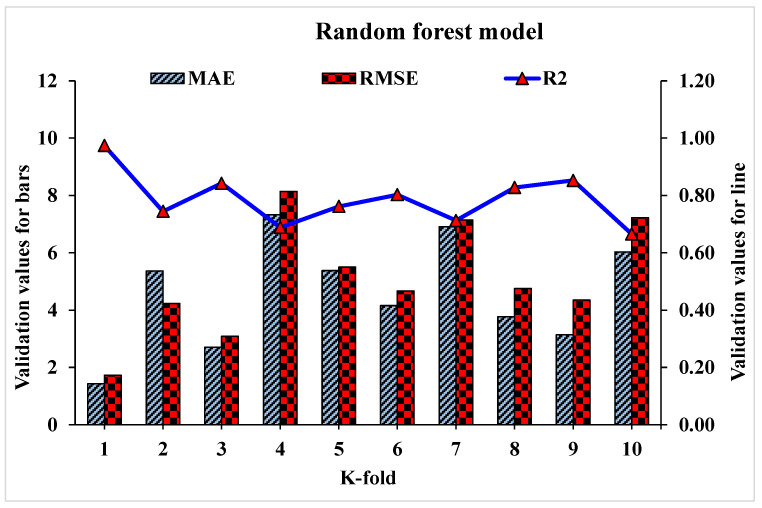
Statistical analysis of RF model for K-fold cross-validation.

**Figure 20:**
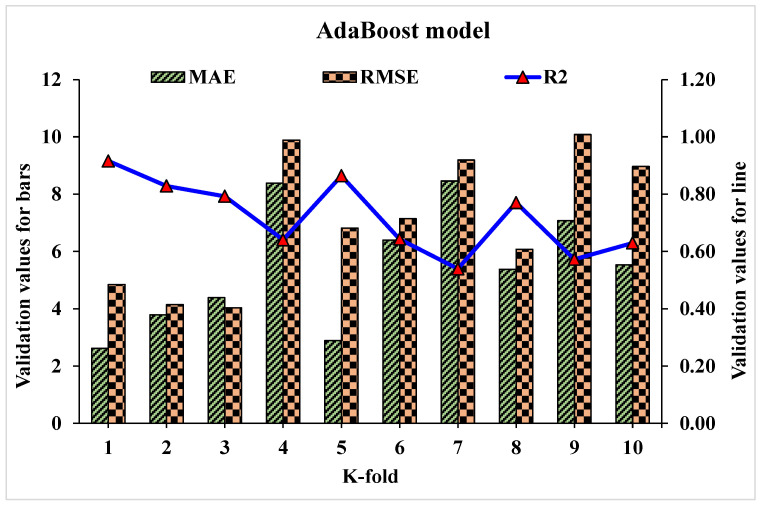
Statistical analysis of AdaBoost model for K-fold cross-validation.

**Figure 21:**
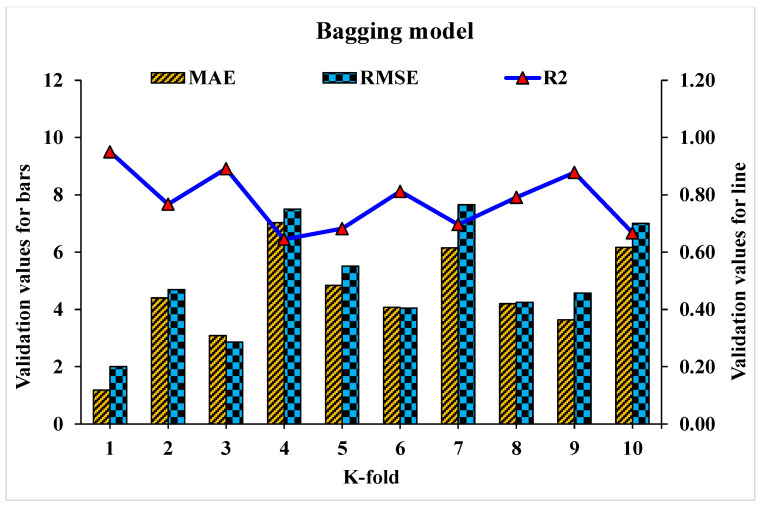
Statistical analysis of bagging model for K-fold cross-validation.

Statistical analysis with Equations (1)–(3) is utilized to predict the model’s response. The model’s legitimacy is evaluated by utilizing the k-fold cross-validation approach during execution [95,96,97]. Usually, the validity of the model is done with a k-fold cross validation process [92], in which random dispersion is done by splitting it into ten groups. The greater the R^2^ value and less the errors (RMSE and MAE), the more a model’s accuracy is. Furthermore, this process should be repeated multiple (i.e., 10) times for a satisfactory result. The exceptional precision of the model can be achieved by using this comprehensive approach. In addition, statistical analysis (i.e., RMSE and MSE) is also performed for all the models (Table 6). The RF model accuracy (inversely related to error values) compared to AdB, Bg, and DT models is also supported by these checks. Statistical analysis, as reported in the literature [98,99,100], is used to assess the model’s response to the prediction. The k fold cross validation is assessed by utilizing R^2^, MSE, and MAE. Respective dispersions for the decision tree, random forest, AdaBoost, and bagging models are presented in Figure 18, Figure 19, Figure 20 and Figure 21. Minimum, average, and maximum values of R^2^ for the decision tree are 0.52, 0.68, and 0.86, respectively (refer to Figure 18). Whereas the maximum, average and minimum values of R^2^ for random forest are 0.97, 0.78, and 0.66, respectively (see Figure 19). Contrary to it, the maximum, minimum, and average R^2^ values of the AdaBoost model are 0.91, 0.53, and 0.71, respectively, as portrayed in Figure 20. The maximum, average, and minimum values of R^2^ for Bg model are 0.95, 0.78, and 0.64, respectively are shown in Figure 21. Upon comparing error values (MSE and MAE), the average MSE and MAE values for DT model are 11.58 and 9.45, respectively. Whereas, average MSE and MAE values for AdaBoost model are 10.08 and 8.45, respectively, and average MSE and MAE values for the Bg model are 7.65 and 7.03, respectively. The RF model with the lowest error and higher R^2^ value performs better for results prediction.

(1) $$\mathrm{MAE}=\frac{1}{n}\overset{n}{\underset{i=1}{\sum }}\left|x_{i}-x\right|$$

(2) $$\mathrm{MSE}=\frac{1}{n}\overset{n}{\underset{i=1}{\sum }}{\left(y_{pred}-y_{ref}\right)}^{2}$$

(3) $$\mathrm{RMSE}=\sqrt{\sum \frac{{\left(y_{pred}-y_{ref}\right)}^{2}}{N}}$$

where:

*n* = Total data samples,

x, $y_{ref}$ = data sample reference values,

$x_{i}$, $y_{pred}$ = model prediction values.

**Table 6:** Statistical checks of decision tree, random forest, AdaBoost, and bagging models.

Models	Mean Absolute Error (MPa)	Mean Square Error (MPa)	Root Mean Square Error (MPa)
Decision tree	6.204	26.173	5.116
Random forest	3.937	24.197	4.919
AdaBoost	4.665	30.947	5.563
Bagging	3.969	62.679	7.917

## 7. Discussion

### 7.1. Comparison of Machine Learning Models

**Figure 22:**
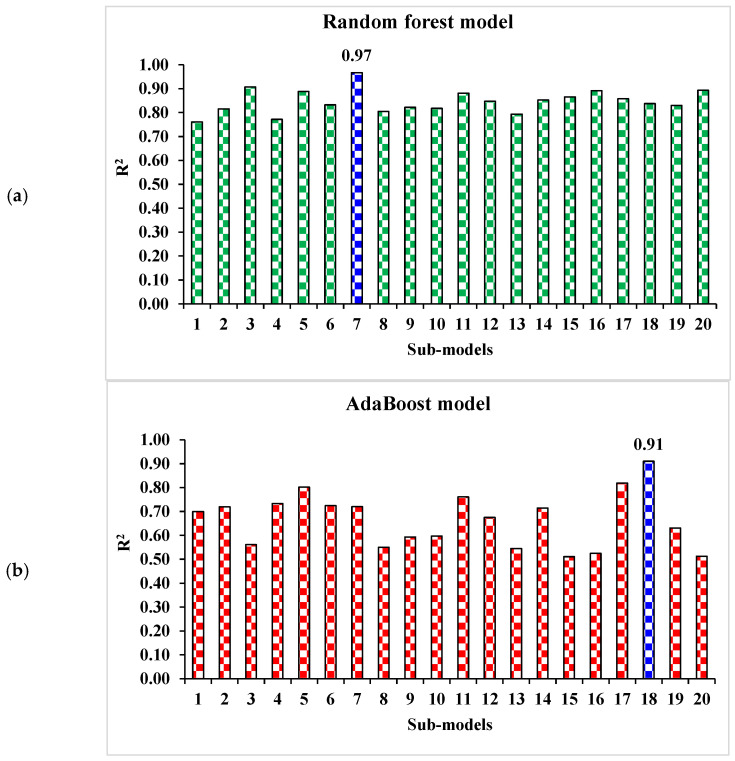

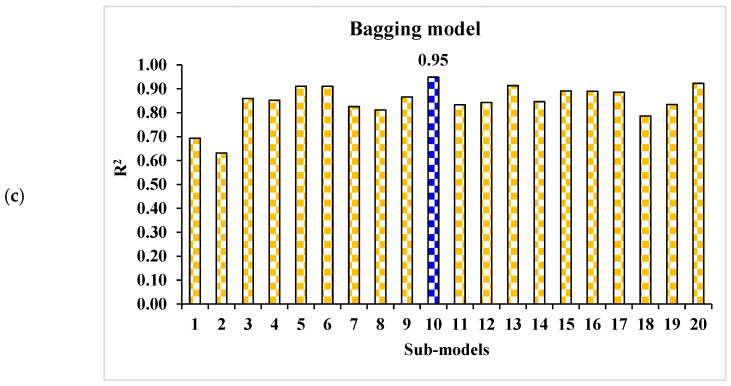
Determination coefficient (R^2^) values for sub-models. (**a**) Random forest; (**b**) AdaBoost; (**c**) Bagging.

Ensembled ML and individual approaches are explored in this study to estimate WMC with the aim of sustainable development in environment-friendly construction materials. RF, Bg, AdB, and DT machine learning techniques are used in this study to predict the compressive strength of WMC. The DT algorithm’s goal is to develop a model that can predict the target variable accurately, for which a tree like structure, i.e., a decision tree, is developed for problem-solving. In DT, the class label is represented by a leaf node and attributes are represented by interior node. Both variance and bias are reduced by boosting supervised learning. Learners develop this idea sequentially on which it is based. The growth of all subsequent learners is based on prior learners, except for the initial one. In this way, strong learners are formed from weak ones. Whereas, in bagging technique, a random sample is selected for data from the training set; i.e., the selection of individual data points can be made multiple times. Individual training of said weak models is done in pursuance of numerous data samples generation and based on task type like; classification or regression, the average and/or majority of these predictions give an estimate with high accuracy. To establish the algorithm’s prediction superiority, employed algorithms are compared for targeted performance. The output of the random forest model comes out to be more accurate, having a 0.97 R^2^ value, compared to bagging with 0.95 R^2^, AdB with 0.91 R^2^, and DT with 0.86 R^2^. Furthermore, the performance of AdB, RF, DT, and Bg models is also evaluated by utilizing the k-fold cross-validation technique and statistical analysis. The performance of the model is higher with low error levels. But it is tough to assess optimized machine learning regressors to forecast results from a wide range of topics because the model’s performance is very much dependable on data points and the model’s input parameters. On the other hand, in ensemble ML techniques, sub-models are generated to leverage the weak learner that can be optimized and trained on data for achieving the higher value of R^2^. Dispersion of values for the determinant coefficient of AdB, bg, and RF sub-models is shown in Figure 22. The values of R^2^ for all sub-models of RF are greater than 0.76, as shown in Figure 22a, while most values of R^2^ in the case of sub-models for AdB and Bg are less than 0.51 (Figure 22b) and 0.66 (Figure 22c), respectively. It depicts higher accuracy of RF technique for results prediction having a maximum value of R^2^, i.e., 0.97. Therefore, the RF model is suggested to predict the compressive strength of waste materials such as marble powder.

### 7.2. Waste Marble Concrete for Sustainable Construction

**Figure 23:**
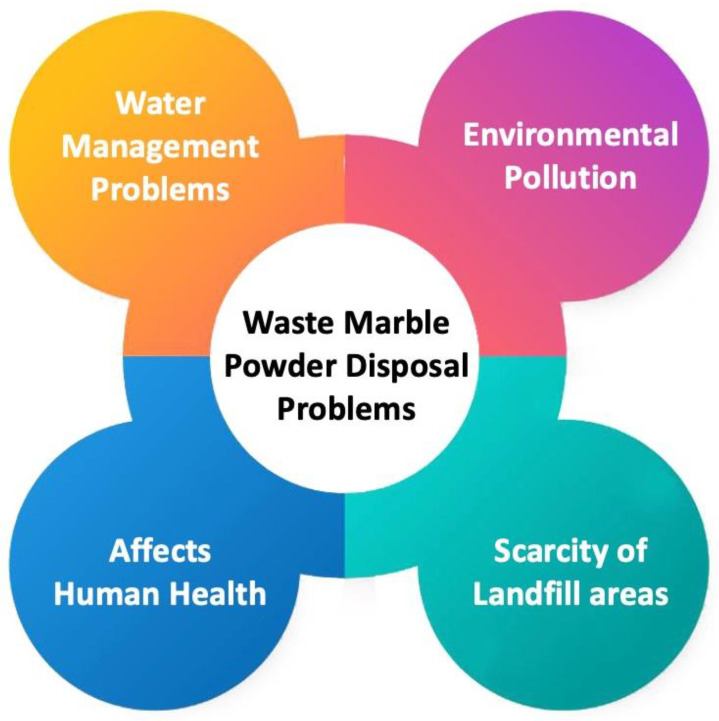
Marble powder disposal problems.

Planet earth is facing destruction of the ecosystem in terms of ground contamination, water pollution, and air quality. These are the leading causes of severe diseases leading to mortality. In addition to health issues, pollution is also the main hindrance to achieving sustainability. A substantial expense for society and the economy is imposed by high levels of environmental pollution, i.e., air, water, and land treatment. Construction wastes are a major contributor to environmental pollution. Singh, et al. [101] reported that 30% of marble is wasted during processing because of its uneven shape or smaller size. In the case of semi-processed slabs, the quantity of waste is 2–5%. In a vertical/horizontal cutter, one ton of processed marble stone produces nearly one ton of slurry with 35–45% water content. Construction industries are expanding too quickly, resulting in a massive amount of waste, wreaking havoc on the environment in terms of air pollution, water pollution, and soil deterioration, such as waste generated by marble industries. To address this major challenge, strong strategy action is required. Researchers/engineers are more focused on the effective usage of waste materials in the construction industry to minimize the challenge mentioned above. The incorporation of waste materials, such as marble powder, is among the effective steps toward sustainability as it would not only reduce the impact on the environment, but would also save natural resources and lower the project’s overall cost, ultimately bringing economic value for waste materials. According to this viewpoint, the building sector is the primary focus for the reuse of waste products such as waste marble and granite, natural waste fibers, aggregate, and mortar wastes, etc. These wastes may be used in large-scale concrete production, whereas renewable resources such as natural sand may last longer and minimize cement usage, resulting in more productive fields, lower project costs, and reduced environmental contamination risk. In the current research, waste marble powder usage is pointed out for concrete manufacturing to reduce waste disposal problems as shown in Figure 23. The concrete blocks are mostly used in the interior and the exterior of buildings. Blocks are used for partition as non-load bearing walls when used in frame structures that are constructed with reinforced cement concrete (RCC). The waste marble powder concrete blocks can deliver several flexible choices that can be used to customize one’s home aesthetics with minimum effort. Because of this functionality, concrete blocks allow design ideas for innovation in the street and building floors. Sustainable concrete blocks are readily recyclable, thus reducing the overall cost of building construction, ultimately eliminating potential pollution. Marble powder is added to concrete to make these blocks which can be used in the construction of roadside walkways. C.S of concrete is reduced by adding waste marble powder to it, as reported in the current study, allowing its application in emergency light-weight structures such as shelter homes, hospitals after earthquakes and flooding, and restrooms for passengers on highways and in railway/bus stations. In this scenario, waste marble powder concrete blocks are proposed to be used as sustainable construction material.

## 8. Conclusions

Marble stone waste materials are a major concern for the construction industry. Accordingly, the incorporation of marble waste powder in concrete composite during its manufacturing could be an effective addition to the category of sustainable construction materials and an effective effort to improve the surrounding environment. For this purpose, an approach has been made to use marble powder with different proportions in concrete. Additionally, this study aims to explore the usage of ensembles machine learning (ML) and individual approaches for the prediction of compressive strength (C.S) of waste marble concrete (WMC). Forecasting the compressive strength of waste marble concrete is achieved by utilizing random forest (RF), AdaBoost (AdB), bagging (Bg), and decision tree (DT) techniques. The conclusions are as follows:

- Bricks manufactured of 10% marble powder as a substitute had the highest compressive strengths of 37.8 MPa at 28 days. Such type of waste marble concrete may be used in the form of blocks for emergency light-weight structures such as hospitals and refuge homes during earthquakes and flooding. In this scenario, WMC having a 10% marble powder content (as a substitute) is proposed to be used as construction material.
- The random forest model has come out to be most effective in terms of prediction with respect to AdaBoost, bagging, and decision tree approaches due to higher values of R^2^ with lower error values. Decision tree, random forest, AdaBoost, and bagging models have R^2^ values of 0.86, 0.97, 0.91, and 0.95, respectively. However, the findings of ensembled models (RF, AdaBoost, and bagging) are within an acceptable range.
- Satisfactory outputs of random forest, AdaBoost, and bagging are also demonstrated by the k-fold cross-validation approach and statistical analysis. In addition, the higher performance of the random forest model with respect to the decision tree, AdaBoost, and bagging models is also established through these checks.
- ML can achieve more accurate prediction of material strength properties approaches without putting additional effort and time for sampling, casting, curing, and testing.

This study evaluated the compressive strength of waste marble concrete considering limited mix proportions with limited input parameters. However, in the future, increasing the number of datasheets and importing a substantially higher number of mixtures and considering higher input parameters could result in a better applicable model. As a result, experimental work, field tests, and numerical analysis employing a variety of methodologies should be used to increase the quantity of data points and outcomes in future investigations (e.g., the Monte Carlo simulation).

## Figures and Tables

**Table 1:** Machine learning algorithms in the literature.

Algorithm Name	Notation	Prediction Properties	Year	Waste Material Used	Ref.
Individual (decision tree) and ensemble algorithm (bagging)	DT and Bg	Compressive Strength	2021	FA	[79]
Ensemble modelling (bagging and boosting)	Bg and AdB	Compressive strength	2021	FA	[22]
Individual Algorithms (decision tree)	DT	Chloride Concentration	2021	FA	[18]
Data Envelopment Analysis	DEA	Compressive strength Slump test L-box test V-funnel test	2021	FA	[80]
Multivariate	MV	Compressive strength	2020	Crumb rubber with SF	[81]
Support vector machine	SVM	Slump test L-box test V-funnel test Compressive strength	2020	FA	[82]
Adaptive neuro fuzzy inference system	ANFIS with ANN	Compressive strength	2020	POFA	[83]
Random forest	RF	Compressive strength	2020	-	[84]
Intelligent rule-based enhanced multiclass support vector machine and fuzzy rules	IREMSVM-FR with RSM	Compressive strength	2019	FA	[85]
Random forest	RF	Compressive strength	2019	FA GGBFS FA	[86]
Decision tree	DT	Compressive strength	2021	Ceramic waste	[62]

**Table 2:** Chemical composition of cement and marble powder.

Components Details	Cement	Marble Powder
Calcium Oxide (CaO)	61.81	42.14
Magnesium Oxide (MgO)	1.96	2.77
Silica (SiO_2_)	22.07	0.79
Potassium Oxide (K_2_O)	0.46	0.63
Alumina (Al_2_O_3_)	6.96	2.69
Sodium Oxide (Na_2_O)	0.11	0.61
Iron Oxide (Fe_2_O_3_)	3.62	1.94
Sulfur Trioxide (SO_3_)	2.14	0.042
LOI	1.2	42.28

**Table 3:** Physical properties of sand and aggregates.

Property	Dry Rodded Bulk Density	Bulk Specific Gravity	Moisture Content	Water Absorption	Fineness Modulus	Nominal Maximum Size
	kg/m^3^	-	%	%	-	mm
Sand	1800	2.61	1.57	2	2.72	-
Aggregate	1601	2.51	1.49	1.65	-	25.4
Followed Standards	ASTM C29	ASTM C128/C127		ASTM C566	ASTM C136	-

**Table 4:** Input parameters description analysis.

	Input Variables					
Parameters	Cement (kg/m^3^)	Marble Powder (kg/m^3^)	Sand (kg/m^3^)	Aggregate (kg/m^3^)	W/C Ratio	Days
Mean	484.396	25.4941	618.7	1202.28	0.45045	17.5
Standard Error	9.72503	2.95033	19.5542	33.6188	0.00637	1.18
Median	472.838	17.238	615.264	1116.36	0.43994	17.5
Mode	486.948	0	620.058	1201.29	0.37997	7
Standard Deviation	86.9833	26.3886	174.898	300.695	0.05696	10.56
Range	398.65	70.89	891.174	1091.64	0.28578	21

**Table 5:** Input and output variables range.

	Parameters	Abbreviation	Unit	Minimum Value	Maximum Value
Input	Cement	C	kg/m^3^	310.148	708.798
	Marble powder	MP	kg/m^3^	0	70.89
	Sand	S	kg/m^3^	129.472	1020.65
	Aggregate	A	kg/m^3^	659.328	1750.97
	Water to cement ratio	W/C	kg/m^3^	0.36273	0.64851
	Days	D	Days	7	28
Output	Compressive Strength	C.S	MPa	9.49	72.11

## Data Availability

The data used in this research has been properly cited and reported in the main text.

## Supplementary Materials

The following supporting information can be downloaded at: https://www.mdpi.com/article/10.3390/ma15124108/s1, Table S1: Data used for modeling.
