# Supplementary material for: Exploring the Use of Waste Marble Powder in Concrete and Predicting Its Strength with Different Advanced Algorithms
Source: Materials (Basel). 2022 Jun 9;15(12):4108. doi: 10.3390/ma15124108 (PMC9227983; doi:10.3390/ma15124108)
Supplement: Supplementary file 1 [file materials-15-04108-s001.zip › materials-1745943-supplementary.pdf]

Supplementary data:

Table S1. Mix proportion

Mix	Cement (kg/m^3)	Marble powder (kg/m^3)	Sand (kg/m^3)	Aggregate (kg/m^3)	W/C ratio	Days	C.S (MPa)
**M1**	486.95	0.00	458.49	1201.29	0.38	7	27.00
**M2**	344.62	0.00	129.47	659.33	0.38	7	33.65
**M3**	407.76	0.00	466.21	1010.07	0.58	7	22.80
**M4**	441.08	0.00	453.39	825.86	0.40	7	21.53
**M5**	526.56	0.00	552.87	1153.14	0.44	7	24.50
**M6**	642.60	0.00	610.47	1079.57	0.47	7	23.07
**M7**	519.52	0.00	592.25	1220.87	0.44	7	30.83
**M8**	501.64	0.00	531.73	923.03	0.44	7	23.50
**M9**	593.54	0.00	730.05	1489.81	0.46	7	25.73
**M10**	598.13	0.00	699.79	1028.74	0.38	7	27.30
**M11**	492.12	0.00	620.06	900.59	0.42	7	30.63
**M12**	599.66	0.00	725.56	1750.97	0.40	7	22.50
**M13**	570.32	0.00	815.56	1608.34	0.44	7	24.07
**M14**	454.72	0.00	600.20	1073.11	0.41	7	23.93
**M15**	444.31	0.00	599.83	1417.36	0.44	7	25.74
**M16**	498.44	0.00	712.74	1006.81	0.39	7	26.00
**M17**	708.80	0.00	1020.65	1672.77	0.38	7	27.90
**M18**	421.06	0.00	627.37	1440.00	0.48	7	34.13

**M19**	524.18	0.00	807.26	1567.33	0.42	7	28.47
**M20**	421.80	0.00	620.06	1016.57	0.36	7	33.90
**M21**	438.26	48.69	458.49	1201.29	0.42	7	28.50
**M22**	310.15	34.48	129.47	659.33	0.42	7	29.80
**M23**	367.00	40.77	466.21	1010.07	0.65	7	13.87
**M24**	396.98	44.10	453.39	825.86	0.44	7	27.30
**M25**	473.89	52.67	552.87	1153.14	0.49	7	24.87
**M26**	578.34	64.26	610.47	1079.57	0.53	7	20.77
**M27**	467.57	51.95	592.25	1220.87	0.49	7	22.43
**M28**	451.49	50.15	531.73	923.03	0.49	7	25.43
**M29**	534.21	59.36	730.05	1489.81	0.51	7	21.70
**M30**	538.29	59.81	699.79	1028.74	0.43	7	31.07
**M31**	442.92	49.20	620.06	900.59	0.47	7	22.23
**M32**	539.68	59.98	725.56	1750.97	0.44	7	22.73
**M33**	513.30	57.02	815.56	1608.34	0.49	7	14.67
**M34**	409.22	45.46	600.20	1073.11	0.46	7	20.10
**M35**	399.87	44.44	599.83	1417.36	0.49	7	15.77
**M36**	448.60	49.84	712.74	1006.81	0.44	7	15.33
**M37**	637.91	70.89	1020.65	1672.77	0.43	7	17.10
**M38**	378.96	42.09	627.37	1440.00	0.54	7	17.70
**M39**	471.78	52.43	807.26	1567.33	0.47	7	18.83

**M40**	379.64	42.19	620.06	1016.57	0.40	7	18.27
**M1**	486.95	0.00	458.49	1201.29	0.38	28	33.67
**M2**	344.62	0.00	129.47	659.33	0.38	28	34.35
**M3**	407.76	0.00	466.21	1010.07	0.58	28	26.77
**M4**	441.08	0.00	453.39	825.86	0.40	28	30.70
**M5**	526.56	0.00	552.87	1153.14	0.44	28	26.23
**M6**	642.60	0.00	610.47	1079.57	0.47	28	27.37
**M7**	519.52	0.00	592.25	1220.87	0.44	28	27.78
**M8**	501.64	0.00	531.73	923.03	0.44	28	36.47
**M9**	593.54	0.00	730.05	1489.81	0.46	28	34.87
**M10**	598.13	0.00	699.79	1028.74	0.38	28	32.60
**M11**	492.12	0.00	620.06	900.59	0.42	28	34.13
**M12**	599.66	0.00	725.56	1750.97	0.40	28	28.03
**M13**	570.32	0.00	815.56	1608.34	0.44	28	26.80
**M14**	454.72	0.00	600.20	1073.11	0.41	28	33.20
**M15**	444.31	0.00	599.83	1417.36	0.44	28	34.87
**M16**	498.44	0.00	712.74	1006.81	0.39	28	37.87
**M17**	708.80	0.00	1020.65	1672.77	0.38	28	36.00
**M18**	421.06	0.00	627.37	1440.00	0.48	28	41.03
**M19**	524.18	0.00	807.26	1567.33	0.42	28	34.77
**M20**	421.80	0.00	620.06	1016.57	0.36	28	32.27

**M21**	438.26	48.69	458.49	1201.29	0.42	28	37.83
**M22**	310.15	34.48	129.47	659.33	0.42	28	33.35
**M23**	367.00	40.77	466.21	1010.07	0.65	28	19.27
**M24**	396.98	44.10	453.39	825.86	0.44	28	28.93
**M25**	473.89	52.67	552.87	1153.14	0.49	28	29.63
**M26**	578.34	64.26	610.47	1079.57	0.53	28	21.33
**M27**	467.57	51.95	592.25	1220.87	0.49	28	27.40
**M28**	451.49	50.15	531.73	923.03	0.49	28	29.77
**M29**	534.21	59.36	730.05	1489.81	0.51	28	29.90
**M30**	538.29	59.81	699.79	1028.74	0.43	28	36.23
**M31**	442.92	49.20	620.06	900.59	0.47	28	27.33
**M32**	539.68	59.98	725.56	1750.97	0.44	28	34.00
**M33**	513.30	57.02	815.56	1608.34	0.49	28	19.23
**M34**	409.22	45.46	600.20	1073.11	0.46	28	24.43
**M35**	399.87	44.44	599.83	1417.36	0.49	28	18.50
**M36**	448.60	49.84	712.74	1006.81	0.44	28	23.90
**M37**	637.91	70.89	1020.65	1672.77	0.43	28	22.30
**M38**	378.96	42.09	627.37	1440.00	0.54	28	23.30
**M39**	471.78	52.43	807.26	1567.33	0.47	28	22.73
**M40**	379.64	42.19	620.06	1016.57	0.40	28	26.23
